# dbOTU3: A new implementation of distribution-based OTU calling

**DOI:** 10.1371/journal.pone.0176335

**Published:** 2017-05-04

**Authors:** Scott W. Olesen, Claire Duvallet, Eric J. Alm

**Affiliations:** 1 Department of Biological Engineering, Massachusetts Institute of Technology, Cambridge, Massachusetts, United States of America; 2 Center for Microbiome Informatics and Therapeutics, Institute for Medical Engineering and Science, Cambridge, Massachusetts, United States of America; National Cancer Institute, UNITED STATES

## Abstract

Distribution-based operational taxonomic unit-calling (dbOTU) improves on other approaches by incorporating information about the input sequences’ distribution across samples. Previous implementations of dbOTU presented challenges for users. Here we introduce and evaluate a new implementation of dbOTU that is faster and more user-friendly. We show that this new implementation has theoretical and practical improvements over previous implementations of dbOTU, making the algorithm more accessible to microbial ecology and biomedical researchers.

## Introduction

Preheim *et al.* [1] formulated the distribution-based OTU-calling (dbOTU) algorithm, an extremely accurate algorithm for grouping DNA sequences from microbial communities into operational taxonomic units (OTUs) for ecological or biomedical research. Unlike most OTU-calling approaches, which group sequences based only on the similarities of the sequences themselves, this algorithm also uses information about the distribution of sequences across samples. This allows dbOTU to distinguish ecologically-distinct but sequence-similar organisms or populations.

### The algorithm

#### Motivation

The algorithm aims to separate genetically-similar sequences that appear to be ecologically distinct (or, conversely, to join less genetically-similar sequences that appear to be ecologically identical). For example, if two sequences differ by only one nucleotide, an OTU-calling algorithm would likely group those two sequences into the same OTU. However, if the two sequences never appeared together in the same sample, an observer would probably conclude that that one nucleotide difference corresponds to two distinct groups of organisms, one which lives in one group of samples, the other living in the other.

Conversely, if two sequences differed by a few nucleotides, an OTU-calling algorithm would probably place those two sequences into different OTUs. However, if the two sequences appeared in the same ratio in all samples (e.g., sequence 2 was always almost exactly ten times less abundant than sequence 1), an observer might conclude that the second sequence was either sequencing error or a member of the same ecological population as the first sequence.

#### The original workflow

The workflow of the original dbOTU implementation was:

1. Process 16S rRNA data up to dereplicated sequences.
2. Create a table of sequence counts showing the number of times each sequence appears in each sample.
3. Align the dereplicated sequences. Using the alignment, make a phylogenetic tree and a “distance matrix” showing the genetic dissimilarity between sequences.
4. Feed the matrix and the table of sequence counts into the algorithm proper, which groups the sequences into OTUs.

#### Overview of the algorithm

The pipeline’s last step is the dbOTU algorithm proper and was the main contribution of Preheim *et al.* [1]. The algorithm is summarized in Fig 2 of that publication. In outline, the algorithm was:

1. Make the most abundant sequence an OTU.
2. For each sequence (in order of decreasing abundance), find the set of OTUs that meet “abundance” and “genetic” criteria. The abundance criterion requires that the candidate sequence be some fold less abundant than the OTU (e.g., so that it can be considered sequencing error). The genetic criterion requires that the candidate sequence be sufficiently similar to the OTU’s sequence (e.g., so that it can be considered sequencing error or part of the same population of organisms).
3. If no OTUs meet these two criteria, make the candidate sequence into a new OTU.
4. If OTUs do meet these criteria, then, starting with the most genetically-similar OTU, check if the candidate sequence is distributed differently among the samples than that OTU. If the distributions are sufficiently similar, merge the candidate sequence into that OTU. Specifically, add the candidate sequence’s counts across samples to the OTU’s counts.
5. If the candidate sequence does not have a distribution across sample sufficiently similar to an existing OTU, then make this sequence a new OTU.
6. Move on to the next candidate sequence.

Note that an OTU’s counts change every time a candidate sequence is merged into that OTU, but an OTU’s sequence never changes. In other words, an OTU’s candidate sequence is the sequence of its most abundant member.

Variations on the dbOTU algorithm are characterized mainly by their choice of genetic criterion, which determines which sequences are sufficiently genetically similar that they are allowed to merged into the same OTU, and distribution criterion, which determines which sequences are distributed sufficiently similarly across samples to be allowed to be merged.

### Previous implementations

The dbOTU algorithm has been implemented twice. Here we will introduce a third implementation. The implementations vary in terms of:

- the exact input files they require,
- how they evaluate the genetic (i.e., sequence similarity) criterion,
- how they evaluate the distribution (i.e., ecological similarity) criterion, and
- the details of the software itself.

These differences are summarized in Table 1.

**Table 1:** Comparison of the dbOTU implementations.

Implementation	Programming languages	Required input	Genetic criterion	Distribution criterion
dbOTU1	Perl, R	Matrices of genetic distances, sequence count table	Values from input genetic distance matrix*	Simulated *χ*^2^ test
dbOTU2	Python 2, R	Unaligned and aligned sequences, sequence count table	Proportion of mismatched sites*	Simulated *χ*^2^ test
dbOTU3	Python 2/3	Unaligned sequences, sequence count table	Levenshtein edit distance	Likelihood-ratio test

*The first two implementations recommended inputting information about dissimilarity of aligned and unaligned sequences, and the dissimilarity used in the genetic criterion was the minimum of those two dissimilarities for each pair of sequences.

#### The first implementation

The original implementation (github.com/spacocha/Distribution-based-clustering) which we term “dbOTU1”, was coded in Perl and R (with accessory shell scripts) and took matrices of genetic dissimilarities and a table of sequence counts as input. The table of sequence counts, similar to an OTU table, countained information about how many times each unique sequence appeared in each sample.

The algorithm performed best when it was provided with two dissimilarity matrices, one computed from unaligned sequences and one computed from aligned sequences. To perform the alignment, the original publication recommended the align.seqs command in mothur [2]. To compute the dissimilarities matrices, the publication recommended the -makematrix option in FastTree [3]. The values in FastTree’s dissimilarity matrix are Jukes-Cantor distances $-\frac{3}{4}\log\left(1-\frac{4d}{3}\right)$, where *d* is the proportion of mismatched sites, that is, the number of mismatches in the aligned sequences divided by the length of the sequences. (If the sequences are aligned and contain gaps, the gaps are not counted in the denominator.)

The genetic criterion in dbOTU1 was articulated as the minimum of the aligned and unaligned Jukes-Cantor distances, which was a work-around for the fact that using the aligned sequences sometimes led to a greater distance between two sequences than would be computed by comparing the unaligned sequences.

The distribution criterion was evaluated using the *p*-value from the *χ*^2^ test of independence as implemented in the chisq.test function in R [4], which was called in a separate process from a Perl script. The *p*-value was compared against a threshold for determining whether two sequences should be merged into one OTU. If the *p*-value fell below some treshold, the distributions of the two sequences across the samples were considered too distinct for the two sequences to be merged. (Because the *p*-values were used as an operational threshold for merging OTUs—rather than as explicit statements about the statistical significance of the similarity of two sequences’ distributions—these *p*-values were never subjected to multiple hypothesis correction.)

Many of the comparisons involved sequences with small numbers of counts, for which the analytical, asymptotic calculation of the *p*-value of a *χ*^2^ test is not accurate. This implementation therefore used a simulated *p*-value, available through the R command’s simulate.p.value option, with 10^4^ simulated contingency tables. This empirical calculation was slow and was the principal bottleneck in running dbOTU1.

#### The second implementation

The second implementation (github.com/spacocha/dbOTUcaller), which we call “dbOTU2”, was coded in Python 2 and interfaced with R using rpy2 (rpy2.bitbucket.org) (with accessory Perl scripts). It took unaligned sequences, aligned sequences, and the sequence count table as input.

Like dbOTU1, dbOTU2 used the minimum of the aligned and unaligned genetic dissimilarities for its genetic criterion. Unlike dbOTU1, which used the Jukes-Cantor distance, dbOTU2 used the simple proportion *d* of mismatched sites and computed these dissimilarities directly from the sequence files only when required by the algorithm. This selective computation avoided loading the matrix of *N*^2^ pairwise distances. Hereafter we refer to this metric, the minimum of aligned and unaligned proportion of mismatched sites, as the dbOTU2 metric.

Like the first implementation, this one used the simulated p-value from R’s chisq.test for its distribution criterion. Unlike dbOTU1, dbOTU2 called the test via rpy2 from the Python script. This removed the need for reading and writing temporary files, but it was still slow and required both R and Python.

### This implementation

This implementation, dbOTU3, aims to improve speed and ease of use. It is written in pure Python and is compatible with Python 2 and 3. For the genetic criterion, this implementation uses the Levenshtein edit distance, the number of single-position insertions, deletions, or substitutions required to transform one sequence into another, as an approximation for the sequence dissimilarity. (The Levenshtein distance is implemented in the python-Levenshtein package; github.com/ztane/python-Levenshtein.) For the distribution criterion, this implementation uses a likelihood-ratio test. The merit of these choices for the genetic and distribution criteria are discussed in the Results.

## Methods

### New genetic and distribution criteria

This implementation evaluates the genetic criterion using the Levenshtein edit distance: a candidate sequence will not be merged into an OTU if 2*E*/(*ℓ*_1_ + *ℓ*_2_) is greater than some threshold, where *E* is the Levenshtein edit distance, *ℓ*_1_ is the length of the candidate sequence, and *ℓ*_2_ is the length of the OTU’s sequence. As shown in the Results, this metric is a good approximation of the proportion of mismatched sites in an alignment.

This implementation evaluates the distribution criterion using the *p*-value from a likelihood-ratio test. Define $x_{1}^{\left(i\right)}$ as the number of counts that the OTU has in sample *i* and $x_{2}^{\left(i\right)}$ as the number of counts the candidate sequence has. Define also $X_{1}=\sum_{i}x_{1}^{\left(i\right)}$ and similarly *X*_2_.

The alternative hypothesis for this test is that the OTU and candidate sequence are distributed “differently”, that is, that each of the $x_{1}^{\left(i\right)}$ and $x_{2}^{\left(i\right)}$ are drawn from different random variables, each of which we model as Poisson-distributed [5]. Thus, the alternative hypothesis is

$$H_{1}:x_{1}^{\left(i\right)}∼\text{Poisson}\left(\lambda_{1}^{\left(i\right)}\right)\;\text{and}\;x_{2}^{\left(i\right)}∼\text{Poisson}\left(\lambda_{2}^{\left(i\right)}\right),$$ (1)

where there are no constraints on the Poisson parameters.

The null model asserts that the OTU and candidate sequence are distributed “the same”, that is, that the candidate sequence’s counts in each sample is drawn from a Poisson random variable whose parameter is proportional to the parameter of the OTU’s Poisson variables, where the constant of proportionality is the same across samples. Specifically, the null model is

$$H_{0}:x_{1}^{\left(i\right)}∼\text{Poisson}\left(\lambda^{\left(i\right)}\right)\;\text{and}\;x_{2}^{\left(i\right)}∼\text{Poisson}\left(\rho \lambda^{\left(i\right)}\right),$$ (2)

We expect that 0 < *ρ* < 1 because the candidate sequence is overall less abundant than the OTU. Asserting maximum likelihood for each model shows that

$$\lambda_{1}^{\left(i\right)}=x_{1}^{\left(i\right)}$$ (3)

$$\lambda_{2}^{\left(i\right)}=x_{2}^{\left(i\right)}$$ (4)

$$\rho =\frac{X_{2}}{X_{1}}$$ (5)

$$\lambda^{\left(i\right)}=\frac{X_{1}}{X_{1}+X_{2}}\left(x_{1}^{\left(i\right)}+x_{2}^{\left(i\right)}\right)$$ (6)

so that the test statistic is

$$\Lambda =-2\left(L_{1}-L_{0}\right)=-2\left[f\left(x_{1}+x_{2}\right)-f\left(x_{1}\right)-f\left(x_{2}\right)\right],$$ (7)

where $L_{1}$ is the likelihood of the alternative model, $L_{0}$ is the likelihood of the null model, $x_{i}=\left\{x_{i}^{\left(1\right)},x_{i}^{\left(2\right)},\ldots \right\}$ and

$$f\left(y\right)=\sum_{i}y^{\left(i\right)}\ln y^{\left(i\right)}-\left(\sum_{i}y^{\left(i\right)}\right)\ln\left(\sum_{i}y^{\left(i\right)}\right).$$ (8)

### Accuracy and speed of new implementation

To evaluate the performance of the new implementation, we compared the results of calling OTUs with dbOTU3, dbOTU2, and with UPARSE [6]. We used the Turnbaugh mock community data set [7] analyzed in the original dbOTU publication.

To prepare the data for input into the OTU callers, we first downloaded the data from gordonlab.wustl.edu/TurnbaughSE_2_10/PNAS_2010.html, which included:

- Mock_clean.fna: quality-screened sequences from all 6 mock communities
- Mock_nochimeras.fna: quality-screened, de-noised, and non-chimeric sequences.

We trimmed all sequences in Mock_nochimeras.fna to 187 nucleotides, the length of the shortest sequence in that file. To align the sequences in Mock_clean.fna with those in Mock_nochimeras.fna, we trimmed the first 14 nucleotides from each sequence in Mock_clean.fna and then trimmed the remaining sequences to 187 nucleotides.

To generate a table of sequence counts, we dereplicated the unique trimmed sequences from Mock_nochimeras.fna. For each sequence in Mock_clean.fna, we checked if that sequence appeared among the unique sequences. If so, we counted it as present in the sample corresponding to the metadata for that sequence. The dereplicated sequences and table of sequence counts are in the Supporting Information (S1 and S2 Files).

To generate the aligned sequences required for dbOTU2, we aligned the unique sequences using mothur’s align.seqs command.

dbOTU2 and dbOTU3 were run with a genetic dissimilarity threshold of 0.1 and with *p* = 0.001 as the distribution criterion threshold. dbOTU2 was run using the aligned and unaligned sequences. UPARSE OTUs were called using usearch -cluster_otus and the same list of unique sequences (but with the summary “size” information required by usearch) at similarity thresholds of 95%, 97%, and 100%. Notably, UPARSE includes chimera checking, which identified chimeras among the sequences from Mock_nochimeras.fna. The specific sequences that UPARSE identified as chimeras depended on the clustering similarity threshold.

We compared the results of the OTU callers with the true composition of each mock community sample (Table S3 in ref. [7]). To link the sequence data with the true mock community composition, which is expressed in terms of the abundances of input isolates, we identified, for each dereplicated sequence, the most genetically-similar reference sequence in MockIsolatesV2.fna, specifically, the one with the smallest proportion *d* of unaligned mismatched sites (S3 File). To compare compositions, we combined the abundance data from all the dereplicated sequences that mapped to the same species.

To benchmark the speed of the new implementation, the three implementations were run on the mock community data using a personal computer.

### Accuracy and speed of the new genetic criterion

When dbOTU3 was run on the mock community data, the genetic criterion was evaluated for 3,688 pairs of sequences. We used those pairs of sequences to benchmark the accuracy and speed of the new genetic criterion.

To evaluate the performance of the Levenshtein metric, we compared the dissimilarities computed by it, by the dbOTU2 metric, and by a gold standard—pairwise alignment using Clustal Omega [8]—for each of the 3,688 pairs of sequences.

To benchmark the speed of the new genetic criterion relative to other in-Python options, we measured the time required to compute the dissimilarities between these pairs using three methods: the dbOTU3’s Levenshtein metric, Biopython’s pairwise2.align.globalxx [9], and an external call to Clustal Omega. These computations were performed on a personal computer.

### Accuracy and speed of the new distribution criterion

When dbOTU3 was run on the mock community data, the distribution criterion was evaluated for 47 OTU/sequence pairs. (In terms of the outlined algorithm in the Introduction, dbOTU3 performed 47 comparisons in step 4.) We used those OTU/sequence pairs to benchmark the accuracy and speed of the new distribution criterion.

To benchmark the speed of the new likelihood-ratio test, we computed the distribution tests for the 47 OTU/sequence pairs using the method of dbOTU1 (i.e., an external call to R’s chisq.test command with 10^4^ simulations), the method of dbOTU2 (the same R command as for dbOTU1 but called using rpy2), and dbOTU3’s likelihood-ratio test.

To evaluate the performance of dbOTU3’s likelihood-ratio test, we compared the *p*-values computed by this new test against a gold standard: the simulated *χ*^2^ test with 10^7^ simulations.

## Results

### The new implementation is faster and performs similarly to the previous one

**Fig 1:**
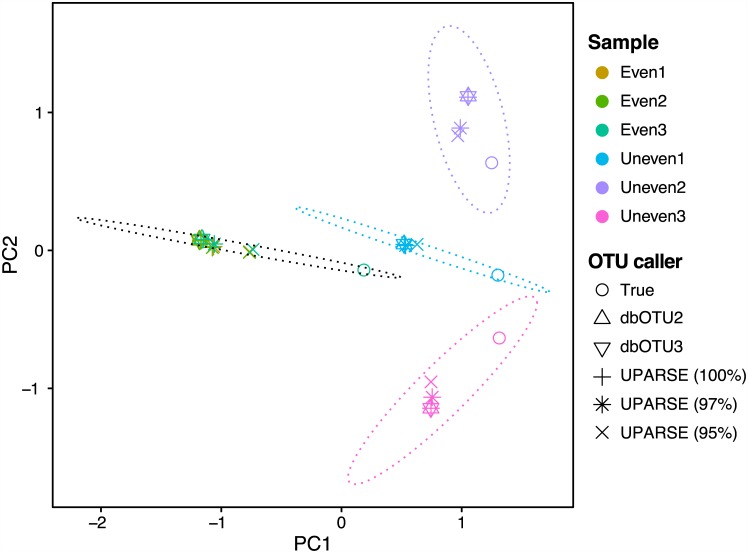
Comparisons of communities analyzed by different methods. dbOTU3 produces nearly identical results with dbOTU2 when visualized in a principal coordinate analysis ordination plot. Each point represents a community resulting from analysis of the mock community data one of the OTU callers. (The two triangles representing dbOTU2 and dbOTU3 always appear on top of one another, making a six-pointed triangle.) The “true composition” is the community composition expected based on how the communities were constructed. The principal components were computed using a matrix of the square roots of the Jensen-Shannon divergence between each pair of computed community compositions.

When processing this relatively small mock community dataset, dbOTU3 was also about ten-fold faster than dbOTU1 and dbOTU2 (Table 2). dbOTU3 also yieled a nearly identical species-level composition when compared to dbOTU2 (Fig 1). Both dbOTU implementations performed similarly to 100% clustering with UPARSE but less similarly to other clustering thresholds with UPARSE.

**Table 2:** Benchmarks for the speed of the entire OTU calling process.

Step	Time (sec)
mothur alignment	3.92 ± 0.34
dbOTU1	13.17 ± 0.73
dbOTU2	13.16 ± 0.17
dbOTU3	1.01 ± 0.01

“mothur alignment” refers to using mothur to align the input sequences, which was required before running dbOTU1 and dbOTU2. The time required to compute the FastTree distance matrix, which is required for dbOTU1, was small (≲0.1 seconds) and is not shown. Errors show the standard deviations over 10 runs.

### The Levenshtein dissimilarity is fast and performs comparably to the previous metric

The Levenshtein genetic dissimilarity was much faster than either in-Python alignments made using Biopython or out-of-Python alignments using Clustal Omega (Table 3).

**Table 3:** Benchmarks for the speed of the dissimilarity metric.

Metric	Time (sec)	Relative time
Biopython	171.3	1119
Clustal Omega	40.3	263
Levenshtein	0.2	1

Times are relative to dbOTU3’s Levenshtein metric. Each method was used to align the 3,688 pairs of sequences that were compared during the evaluation of dbOTU3 on the mock community data.

**Fig 2:**
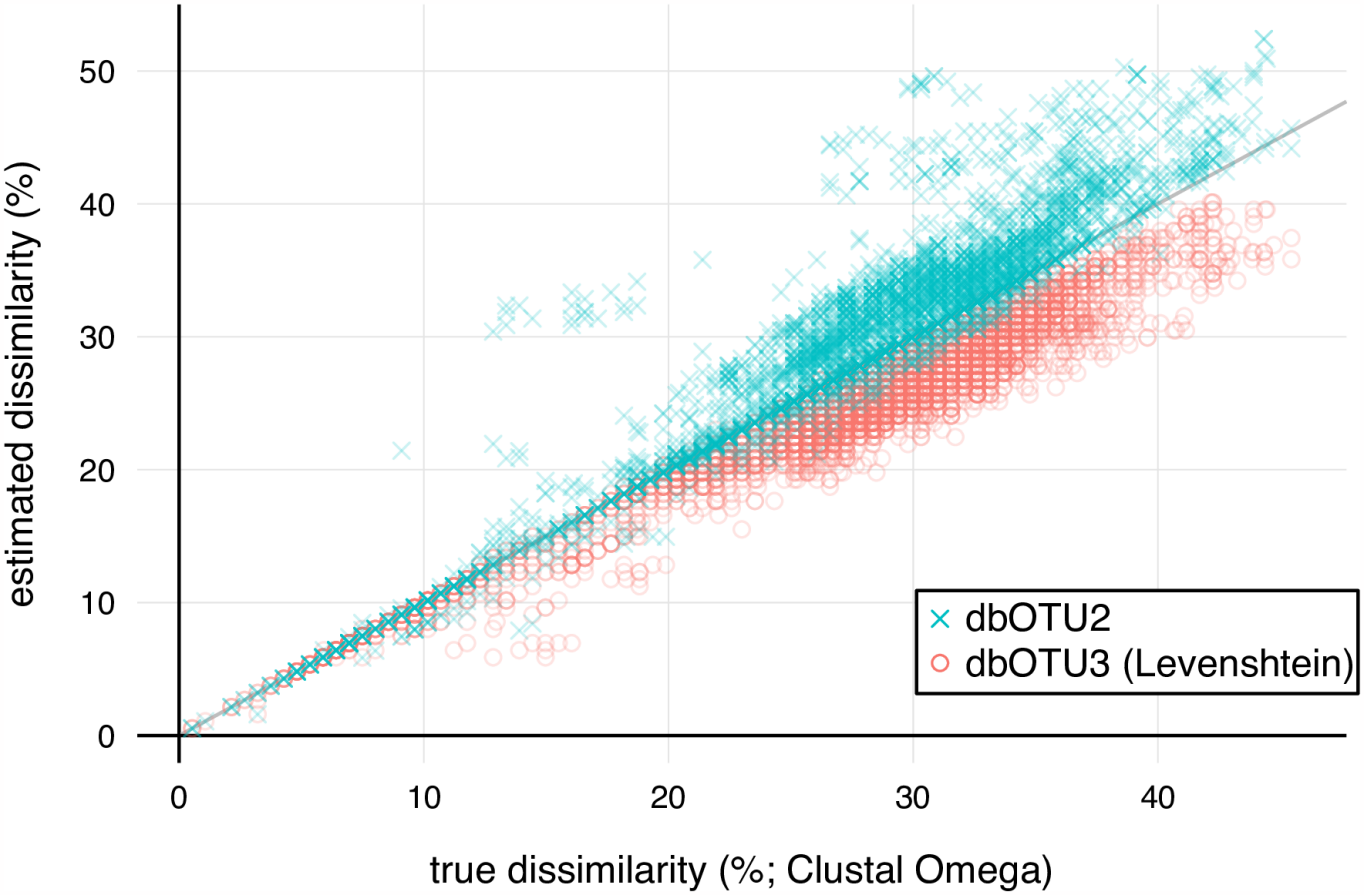
Comparison of genetic dissimilarities. The dbOTU2 metric (blue crosses) and dbOTU3’s Levenshtein metric (pink circles) predict true pairwise dissimilarities. Each point represents a comparison between a pair of sequences that were subjected to the genetic criterion while running dbOTU3 on the mock community data.

The relationship between the genetic dissimilarities measured by the gold standard (the pairwise alignments made with Clustal Omega), the dbOTU2 metric, and dbOTU3’s Levenshtein metric are shown in Fig 2. Both metrics perform well for dissimilarities below 10%. Above 10% true dissimilarity, the Levenshtein metric underestimates dissimilarities, while the dbOTU2 metric mostly overestimates dissimilarities.

To quantify the performance of the new Levenshtein dissimilarity, we separately assessed the two roles the genetic dissimilarity plays in the dbOTU algorithm. First, as laid out in step 2 of the algorithm in Section Overview of the algorithm, the genetic dissimilarity is used to classify OTU/sequence pairs as above or below the genetic criterion threshold: sequences that are too dissimilar to the existing OTUs are immediately made into new OTUs. Second, as laid out in step 4 of the algorithm, sequences that are sufficiently similar to at least one OTU have their distributions compared to those genetically-similar OTUs. The candidate sequence is compared to the most genetically-similar OTU first, then the next-most similar, and so on.

To evaluate the first role of the genetic dissimilarity, we considered its ability to correctly classify sequence pairs as above or below the genetic dissimilarity threshold. We compared the classification performance of dbOTU3’s Levenshtein metric and the dbOTU2 metric against a gold standard, pairwise-alignments by Clustal Omega (Table 4). The dbOTU2 metric is prone to erroneously asserting that two sequences are too dissimilar to be considered for merging, while the dbOTU3 metric is prone to erroneously asserting that two sequences are sufficiently similar to be subject to the distribution test and potentially merged.

**Table 4:** Accuracy of the genetic classification.

Metric	Level (%)	True similars	False similars	False dissimilars	True dissimilars	Sensitivity (%)	Specificity (%)
**dbOTU2**	5	34	0	0	3 654	100.0	100.0
	10	166	11	1	3 510	99.4	99.7
	20	435	3	52	3 198	89.3	99.9
	30	1 623	17	474	1 574	77.4	98.9
**dbOTU3**	5	34	0	0	3 654	100.0	100.0
	10	167	30	0	3 491	100.0	99.1
	20	484	139	3	3 062	99.4	95.7
	30	2 097	854	0	737	100.0	46.3

The genetic similarity metrics used in dbOTU2 and dbOTU3 were used to classify pairs of sequences as sufficiently genetically similar to be subjected to the distribution criterion. To compute the accuracies, dissimilarities for the 3,688 pairs of sequences compared while running dbOTU3 on the mock community data (i.e., the same ones shown in Fig 2) were computed by the gold standard, dbOTU2, and dbOTU3 (Levenshtein) metrics. Here a “similar” result means that a metric concludes that a pair of sequences are sufficiently genetically similar to be considered for merging into an OTU; a “dissimilar” results means that the sequences are too dissimilar to be merged. The “level” is the genetic dissimilarity threshold used for the test. For example, a dbOTU2 true similar at the 5% level means that the dbOTU2 metric and the gold standard both concluded that the two sequences were at least 95% similar and thus candidates for merging. A false dissimilar, by contrast, means that the sequences were genetically similar but the metric concluded they were not, thus erroneously excluding them from a distribution criterion check.

To evaluate the second role of the genetic dissimilarity, we computed the correlation between the dbOTU metric and the gold standard for sequences pairs that passed the genetic criterion at the default 10% dissimilarity threshold. Among these sequence pairs, the Levenshtein metric is slightly better correlated with the true distance than the dbOTU2 metric metric (Table 5).

**Table 5:** Correlation coefficients between the genetic dissimilarities.

Metric	Correlation coefficient (%)
dbOTU2	94.3 (93.9–94.6)
dbOTU3	97.1 (96.9–97.2)

The correlations are between the values computed by the gold standard (pairwise alignment with Clustal Omega) and by the metrics used in dbOTU2 and dbOTU3. The correlations were computed for the 167 pairs of sequences that were compared while running dbOTU3 on the mock community data and for which the true genetic dissimilarity is at most 10% (i.e., those points in Fig 2 for which the true dissimilarity is at most 10%). Ranges are 95% confidence intervals.

### The likelihood-ratio test is fast and mostly reproduces the previous distribution criterion

The likelihood-ratio test used in dbOTU3 was markedly faster than the distribution criteria used in dbOTU1 and dbOTU2 (Table 6).

**Table 6:** Benchmarks for the speed of the distribution criteria.

Distribution criterion	Time (sec)	Relative time
dbOTU1	7.112	963
dbOTU2	0.146	20
dbOTU3	0.007	1

The time reported is the time required to evaluate the distribution criterion for the 47 OTU/sequence pairs that were evaluated while running dbOTU3 on the mock community data.

The likelihood-ratio test also performed similarly to the gold standard (*χ*^2^ test with 10^7^ simulations). Of the 47 OTU/sequence comparisons, the gold standard determined that the OTU and candidate sequence were differently distributed in 16 cases. The likelihood-ratio test concurred in those 16 cases. In one case, shown in Table 7, the gold standard classified the candidate sequence and OTU as similarly-distributed but the likelihood-ratio test classified them as differently-distributed. This performance (16 true dissimilars and 1 false dissimilar) corresponds to an accuracy of *F*_1_ = 97%.

**Table 7:** Sequence count information for the case in which the simulated *χ*^2^ test and likelihood-ratio test disagree.

	Even1	Even2	Even3	Uneven1	Uneven2	Uneven3
seq15	138	129	163	92	258	14
seq45	15	11	28	1	13	1

In this case, the simulated *χ*^2^ test determines that the OTU (top row) and candidate sequence (bottom row) are identically distributed but the likelihood-ratio test determines that they are differently distributed (both with respect to the threshold *p* = 0.001).

Up to this point, the *p*-value threshold for the likelihood-ratio test was fixed at the same value as was used for the gold standard, the simulated *χ*^2^ test. To check if the likelihood-ratio test would perform better when using a different threshold, we varied the likelihood-ratio test’s threshold, keeping the *χ*^2^ test’s threshold fixed, and computed the new test’s accuracy (Table 8). The likelihood-ratio test performs best, relative to the *χ*^2^ test, when its *p*-value threshold is about twenty-fold smaller.

**Table 8:** Likelihood-ratio test at varying *p*-value thresholds.

Threshold *p*-value	True dissimilars	False dissimilars	False similars	Accuracy (*F*_1_; %)
0.0000004	14	0	2	93.3
0.0000020	15	0	1	96.8
0.0000045	16	0	0	100.0
0.0002374	16	1	0	97.0
0.0021540	16	2	0	94.1
0.0024196	16	3	0	91.4
0.0028419	16	4	0	88.9
0.0059160	16	5	0	86.5
0.0241790	16	6	0	84.2
0.0308700	16	7	0	82.1
0.0417458	16	8	0	80.0
0.0436377	16	9	0	78.0
0.0575800	16	10	0	76.2

The likelihood-ratio test perfectly reproduces the results of the *χ*^2^ test for a smaller *p*-value threshold (∼5 × 10^−6^). In these comparisons, the *χ*^2^ test’s *p*-value threshold was fixed at 0.001, and the likelihood-ratio test’s threshold was adjusted to intermediate values appearing in the list of *p*-values the test computed. Here “dissimilar” means that the test returned a *p*-value below the threshold, supporting the conclusion that the OTU and sequence are distributed differently; a “similar” result means that test returned a *p*-value above the threshold, not contradicting the conclusion that the OTU and sequence are distributed similarly. The *χ*^2^ test delivered 16 “dissimilar” results.

## Discussion

This implementation is overall more user-friendly than previous implementations. The software itself is faster, and the codebase is smaller. The main codebase is all in a single file and is supported by online documentation and unit tests. The input files for this implementation are easier for the user to prepare because they do not require sequence alignment.

Aside from software, this implementation has two main differences from the previous implementations. The first is the genetic dissimilarity criterion. Ideally, the genetic dissimilarity metric would be computed by a pairwise alignment of sequences. Unfortunately, there is no efficient implementation of this kind of alignment in pure Python. We found that calling Clustal Omega as a separate process was more than a hundred times slower than computed the Levenshtein distance. If this kind of computation is efficiently implemented in Python (perhaps in scikit-bio; scikit-bio.org), the Levenshtein metric should be replaced. In the meantime, the Levenshtein dissimilarity is efficient and well-supported.

Compared to the dbOTU2 genetic dissimilarity metric, the Levenshtein metric is less likely to erroneously exclude a sequence from being merged into an OTU on the grounds of genetic dissimilarity, but it is conversely more likely to erroneously conclude that a candidate sequence and OTU are genetically similar. dbOTU3 users should treat the genetic threshold as an inclusion criterion rather than an exclusion criterion: sequence pairs more similar than the threshold will almost certainly be treated as similar, but pairs less similar than the threshold can also be expected to be (erroneously) treated as similar and therefore subjected to the distribution criterion.

The second major difference from previous implementations is the distribution criterion. In previous implementations, the simulated *χ*^2^ test, which is computationally demanding, was used in place of the asymptotic *χ*^2^ test, which is not computationally demanding, because the asymptotic *χ*^2^ test is not accurate when some cells have low counts (i.e., the candidate sequence is overall rare or is absent or nearly absent from some samples). This implementation uses a likelihood-ratio test that performed similarly to the simulated *χ*^2^ test when using the same *p*-value threshold, and adjusting the threshold for the likelihood-ratio test to twenty-fold smaller perfectly reproduced the criterion emerging from the *χ*^2^ test. We therefore recommend that users migrating from a previous dbOTU implementation adjust the *p*-value threshold similarly.

In the analysis of the mock community data, these differences in implementation of the genetic and distribution criteria had a negligible effect on the resulting inferred community compositions. Therefore, the degree to which this implementation’s results did not recapitulate the “true” compositions is addressed by the original publication, which rigorously showed that the dbOTU algorithm was more accurate than alternative methods.

## Supporting information

S1 File Unique sequences used for benchmarking. The dereplicated, trimmed sequences from Mock_nochimeras.fna as described in the Methods in fasta format.
(FASTA)

S2 File Table of sequence counts. The columns are the samples in the mock community described in the Methods; the sequences are those that appear in S1 File. Tab-separated.
(TSV)

S3 File Mapping from sequence to isolate. A tab-separated list of sequence IDs (the same that appear in S1 and S2 Files) and the corresponding isolate listed in Table S3 of ref. [7].
(TSV)
